# Enhanced Cellular Delivery and Biocompatibility of a Small Layered Double Hydroxide–Liposome Composite System

**DOI:** 10.3390/pharmaceutics6040584

**Published:** 2014-11-26

**Authors:** Haiyan Dong, Harendra S. Parekh, Zhi Ping Xu

**Affiliations:** 1 Australian Institute of Bioengineering and Nanotechnology, The University of Queensland, Brisbane, QLD 4072, Australia; E-Mail: h.dong@uq.edu.au; 2 Pharmacy Australia Centre of Excellence, School of Pharmacy, The University of Queensland, Brisbane, QLD 4102, Australia

**Keywords:** small layered double hydroxides, liposome, gene delivery, endosomal escape, cytotoxicity

## Abstract

The various classes of gene delivery vectors possess distinct advantages and disadvantages, each of which impacts on cargo loading, delivery and, ultimately, its function. With this in mind, herein we report on a small layered double hydroxide (sLDH)–liposome composite system, drawing upon the salient features of LDH and liposome classes of vectors, while avoiding their inherent shortfalls when used independently. sLDH–liposome composites were prepared by the hydration of freeze-dried matrix method. These composite systems, with a *Z*-average size of ≈200 nm, exhibited low cytotoxicity and demonstrated good suspension stability, both in water and cell culture medium after rehydration. Our studies demonstrate that short dsDNAs/ssDNAs were completely bound and protected in the composite system at an sLDH:DNA mass ratio of 20:1, regardless of the approach to DNA loading. This composite system delivered DNA to HCT-116 cells with ≈3-fold greater efficiency, when compared to sLDH alone. Our findings point towards the sLDH-liposome composite system being an effective and biocompatible gene delivery system.

## 1. Introduction

Gene therapy aims to use genetic materials, namely DNA or RNA, as a therapy; this approach is expected to lead to effective treatment of a wide range of disorders of genetic origin, most of which are not amenable to curative therapy using conventional “small molecule” agents [1,2]. A growing body of evidence points towards the promise of gene therapy as an effective means of treating cancers, as well as genetic and neurodegenerative diseases [3,4]. That said success of gene therapy trials relies heavily on access to safe and efficient vectors that are able to overcome the various extra- and intra-cellular barriers faced by genetic material [5,6,7,8].

Layered double hydroxides (LDHs), otherwise known as anionic clays, are either sourced naturally in the form of minerals or can be synthesised with precise composition and particle size homogeneity [9,10,11,12]. Here, magnesium-aluminium-based LDHs (MgAl–LDH) have proven to be excellent gene delivery vehicles, given their intrinsically low cytotoxicity, good biocompatibility, well-defined particle size and cationic surface properties [13,14,15,16,17,18,19,20]. The positively-charged surface of LDH nanoparticles (NPs) allows ready adsorption of negatively-charged (genetic) material, driven by electrostatic attraction, a phenomenon that leads to cargo being protected from degradation by ubiquitous enzymes, while the net cationic charge of LDH–gene complexes facilitates cellular internalisation. Once internalised, the gradual dissolution of LDH NPs in the acidifying conditions of the endosome results in sustained/controlled release of the payload, which, in turn, raises the osmotic pressure in the endosome and leads to an influx of water, causing swelling and, finally, rupture of endosomal vesicles [21,22]. Once the LDH–gene complexes enter the cytosol, the LDH system is rapidly disassembled, with the Mg and Al ions eliminated through an abundance of membrane-based ion channels. A major drawback to the use of LDH is the mass aggregation of LDH NPs resulting from the interaction with serum proteins abundant in systemic circulation, a feature that has prevented the wider application of LDH as an *in vivo* compatible gene vector.

Separately, liposomes have long been trialled and accepted as effective drug/gene vectors, given their similarities with the cell membrane, both in structure and composition [23]. Moreover, the surface of PEGylated liposomes has been readily modified with specific ligands for targeted delivery [24,25,26,27]. A rate-limiting step with many non-viral vectors, including PEGylated liposomes, is their inability to escape endosomes in a timely manner. Hence, researchers have dedicated their efforts to preparing PEGylated liposomes encompassing various fusogenic lipids (e.g., dioleoylphosphatidyl ethanolamine (DOPE)) and pH-sensitive polymers, such as polyethylenimine (PEI), to improve the endosomal escape and payload release properties [28,29].

More recently, lipid-coated hybrid nanoparticles (NPs), such as polymeric NPs, mesoporous silica NPs and calcium phosphate NPs, have found a place in combination therapy, where attempts are being made to enhance their therapeutic efficacy, while reducing drug resistance and side effects. Such hybrid systems aim to merge the beneficial features from both vectors into one nanocarrier, while mitigating their individual drawbacks [26,30,31,32,33,34]. In particular, Bégu *et al.* prepared layered double hydroxide–liposome hybrid materials with unilamellar liposomes present in the interlayer of the layered double hydroxide by anionic exchange [35], which could be a potential drug storage and sustained release system. Huang *et al.* reported a dextran-magnetic layered double hydroxide-fluorouracil liposome (DMFL) prepared by the reverse evaporation method [36] and claimed that layered double hydroxide nanoparticles were entrapped in the core of liposomal vesicles with the sustained release of fluorouracil. Very recently, Yan *et al.* [37] reported a PEGylated lipid coated LDH delivery system with a core-shell structure with more effective cancer drug delivery.

In this research, we designed a new composite system, comprising of small LDH nanoparticle (30–50 nm) and liposomes, one that would synergistically enhance the colloidal stability and delivery efficiency with reduced side effects. We have demonstrated that our novel LDH–liposome composite system possesses good colloidal stability and high rates of gene transfection with consistent dimensions in the low nanometre size range.

## 2. Materials and Methods

### 2.1. Preparation of LDH NPs and LDH–Liposome Composites

Small LDH (sLDH) and large LDH (L-LDH) NPs were prepared as we reported earlier [38,39]. The LDH-liposome composite system was prepared by the hydration of freeze-dried matrix (HFDM) method, with slight modifications. The minor deviation from the published [40,41,42,43] method involved using 30% *v*/*v* tertiary butyl alcohol (TBA, Sigma–Aldrich, Castle Hill, Australia, ≥99.0%), as opposed to 50% *v*/*v* TBA [44]. Typically, 70 µL of 400 µg/mL sLDH suspension was mixed with 70 µL of 95 mg/mL sucrose solution, followed by further mixing with 60 µL of 450 µg/mL EPC (Egg-derived l-α-lysophosphatidylcholine, Avanti Polar Lipids, Alabaster, AL, USA). The resulting clear TBA/water/EPC three-phase mixture was then snap-frozen in dry ice, followed by freeze-drying for 24 h (Christ Alpha 2-4 LD, Osterode, Germany). Finally, the freeze-dried matrix was hydrated with 50–200 µL water and stood at room temperature for 10 min.

### 2.2. Incorporation of DNA into LDH–Liposome Composite

**Scheme 1:**
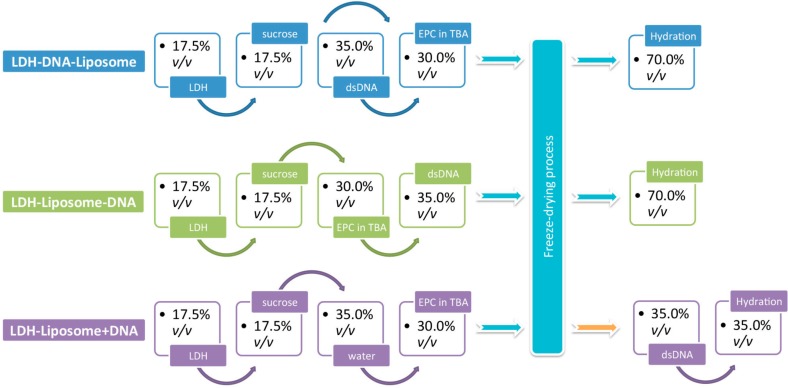
Sequential schematic outlining the preparation of various composite layered double hydroxide (LDH)-liposomal formulations (suppose the total volume of mixed solution was 100% before freeze-drying).

The complexation of DNA with our LDH–liposome composite was trialled using three different approaches, denoted as “LDH–DNA–liposome”, “LDH–liposome–DNA” and “LDH–liposome + DNA”. As depicted in Scheme 1 and elaborated here, the “LDH–DNA–liposome” formulation involved first complexing DNA with LDH in a sucrose solution, followed by mixing in the EPC in TBA solution, then freeze-drying and hydrating the lyophilisate. Similarly, for the “LDH–liposome–DNA” formulation, DNA was added to an LDH suspension containing EPC in TBA, with similar post-treatment. Finally, for “LDH–liposome + DNA”, we first prepared the LDH–liposome composite with DNA added directly to the nanosuspension.

### 2.3. Suspension Stability Test

All particle size distributions were determined by dynamic light scattering (DLS, Zetasizer Nano ZS, Malvern Instruments, Malvern, UK), where LDH, LDH–DNA, LDH–liposome and LDH–liposome with DNA suspensions were diluted with water at a volume ratio of 1:1 before measurement. For their size distribution in cell culture media, they were diluted with complete cell culture media (Gibco^®^ 10% *v*/*v* of foetal bovine serum (Life Technologies, Carlsbad, CA, USA) mixed with Dulbecco’s Modified Eagle Medium (DMEM, with l-glutamine and 4.5 g/L of glucose, Gibco^®^) at a volume ratio of 1:1.

### 2.4. Agarose Gel Electrophoresis

The dsDNA (21 bases, sequencing/PCR purity, GeneWorks, Hindmarsh, Australia) loaded with LDH NPs and LDH–liposome composites was assessed by agarose gel electrophoresis. A 2.5% agarose (molecular grade, Bioline, Alexandria, Australia) gel with Invitrogen™ Gel-Red (Life Technologies, Carlsbad, CA, USA) stain was made, and then dsDNA bound with LDH NPs/LDH–liposome composites was loaded in the wells. For each well, 260 ng dsDNA were used. The gel was imaged by a Bio-Rad imaging system after running at 90 V for 45 min in TBE (Tris/borate/EDTA, Invitrogen™) buffer.

### 2.5. Cell Viability

Human colon cancer HCT-116 cells were seeded in 96-well plates at a density of 2000 cells per well in 200 µL of cell culture media. After 24 h of incubation, cell culture media was replaced by 200 µL of fresh media with the desired concentration of LDH or LDH–liposome NPs. After 48 and 72 h of further incubation, 20 µL of MTT reagent (5 mg/mL in PBS buffer, 3-(4,5-dimethylthiazol-2-yl)-2,5-diphenyltetrazolium bromide, Molecular Probes^®^, Carlsbad, CA, USA) were added, followed by 2–4 h of incubation (dependent on cell density). Next, the cell culture media was discarded, and 50 µL of DMSO (Sigma–Aldrich, Castle Hill, Australia, BioReagent, ≥99.9%) was added to each well, followed by 10 min of incubation. Absorbance was read using a plate reader (Bio-Tek, Winooski, VT, USA) at 540 nm after 1 min of orbital shaking. A negative control group (only cell culture media, without cells) was used as the background. A group without adding NPs was used as the control. The cell viability was normalised to the control. The experiments were conducted for at least two batches in triplicate. Data are presented as the mean ± SEM (standard error of the mean). Two-way ANOVA was used to assess the statistical significance using commercial transfection reagent Oligofectamine™ (Life Technologies, Carlsbad, CA, USA) as the control group.

### 2.6. Cellular Uptake

HCT-116 cells were seeded in 6-well plates at a density of 1 × 10^5^ cells per well in 2 mL complete cell culture media. After 24 h of incubation, cell culture media was replaced with 1 mL of fresh media containing the desired concentration of LDH–DNA–Cy3 NPs (single-stranded DNA, 21 bases, labelled with Cy3, transfection purity, GeneWorks) or LDH–liposomes with DNA–Cy3 NPs (10 and 20 nM DNA). After 4 h of further incubation, the culture media was removed; cells were washed twice with PBS buffer (Gibco^®^) and then detached from the plates by trypsin–EDTA (Gibco^®^). The cells were washed twice with PBS buffer (Gibco^®^) and then fixed in a certain volume of 2% PFA (paraformaldehyde, Chemsupply, Gillman, Australia) before measurement by flow cytometry (BD Accuri™ C6 Flow Cytometer System, BD Biosciences, San Jose, CA, USA; band pass filter 585/40 was used; 10,000 cells were counted). All treatments were performed for three batches in duplicate. Data are presented as the mean ± SEM. Two-way ANOVA was used to assess the statistical significance.

## 3. Results and Discussion

### 3.1. sLDH–Liposome Composite Formation

**Figure 1:**
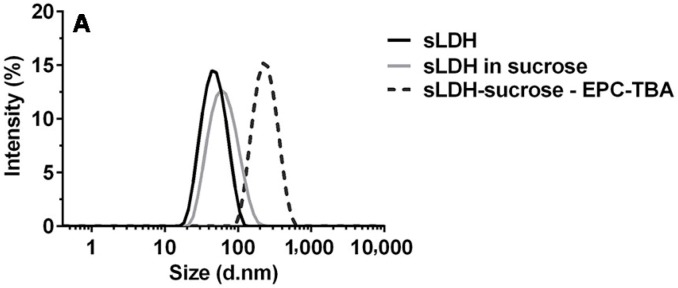

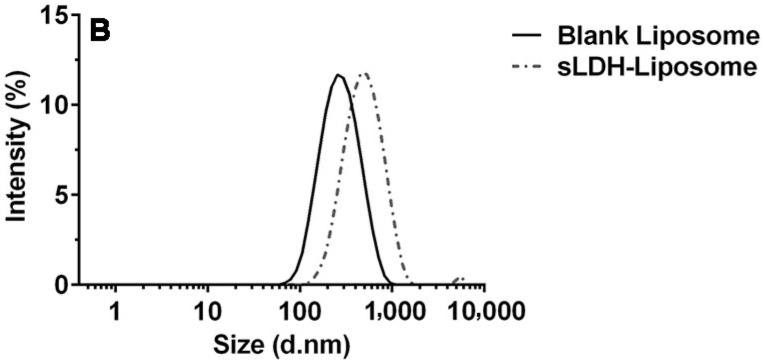
Particle size distribution of small LDH (sLDH) in various mixtures (**A**); blank liposome and sLDH–liposome by the hydration of freeze-dried matrix (HFDM) method (**B**).

**Figure 2:**
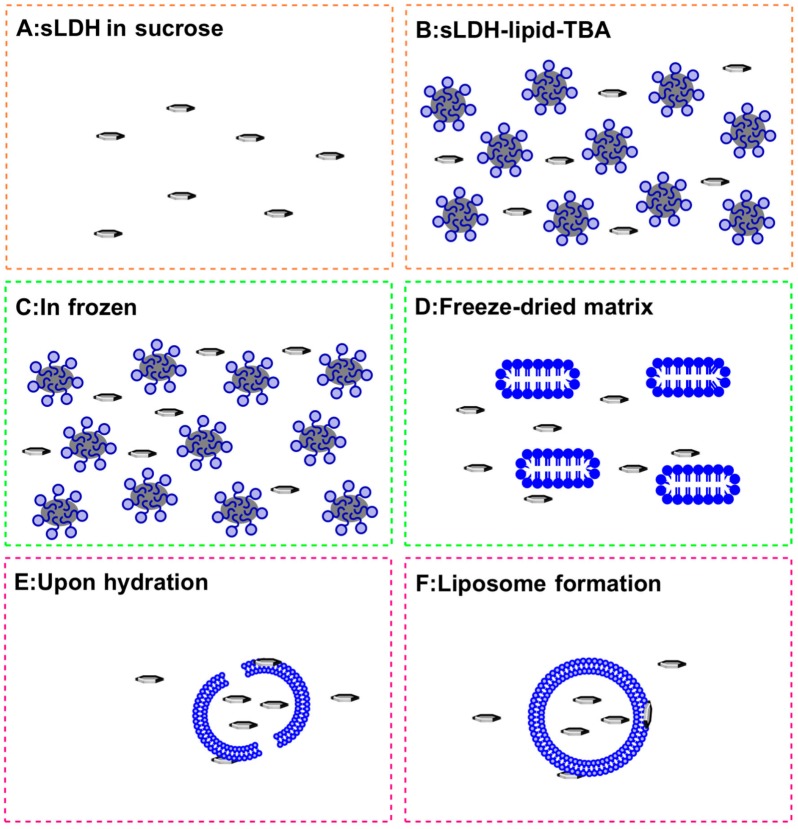
Schematic of the proposed sLDH–liposome composite formation by the HFDM method (for clarity, sucrose is not shown).

As shown in Figure 1A, sLDH possesses a narrow particle size distribution in the range of 10 to 100 nm with a *Z*-average size of ~40 nm. L-LDH NPs have a particle size in the range of 20 to 200 nm with a *Z*-average size of ~100 nm (data not shown). After mixing with sucrose solution, the LDH suspension particle size increased marginally because sucrose lowered the ion strength of the solution [45]. The reduced ion strength may increase the electrostatic double layer around sLDH NP and thus lead to an increased hydrodynamic particle size of sLDH nanoparticles measured by DLS (Figure 1A). While in a homogeneous TBA/water/EPC three-phase mixture, the *Z*-average particle size increased considerably to ~200 nm, indicative of the formation of lipidic micelles (Figure 1A and Figure 2B).

Upon cooling, TBA present within the core of micelles freezes first, given its relatively high freezing point (25.5 °C), this, in turn, freezes the lipid molecules surrounding the TBA core. The water phase freezes next, albeit more gradually due to the presence of sucrose and its high concentration, which further assists with the more uniform distribution of the LDH-payload in the water phase of the mixture (Figure 2C) [43].

Water and TBA are removed completely during the next freeze drying step. During this process, the lipid molecules are expected to re-arrange themselves, forming fragments of lipid bilayers, while sucrose serves to further stabilise these transient structures (Figure 2D). Upon hydration, the lipid bilayer fragments spontaneously assemble and seal, forming liposomes (Figure 2E,F). Due to the relatively small size of sLDH NPs (~40 nm) compared to the forming liposomes, a proportion of the NPs are encapsulated in the vesicles during lipid-fragment self-assembly into liposomes, although one can indeed expect some sLDH NPs to escape encapsulation (Figure 2F). In contrast, when considering this process from the perspective of L-LDH NPs and their capture into forming liposomes (Figure 2F), one would expect significant challenges, stemming from the considerably larger (~100 nm) size of L-LDH (*cf.* sLDH), and so, they are deemed to be less amenable to composite particle formation.

The process leading to the formation of sLDH–liposome composite NPs prepared by the HFDM method is illustrated in Figure 2, with the particle size distribution of blank liposome and the sLDH–liposome shown in Figure 1B. When comparing particle size, the sLDH–liposome composite with a *Z*-average size of ~240 nm was found to be greater than the blank liposome (~200 nm); this difference may be attributed to the adsorption of sLDH NPs onto the surface of liposomal vesicles (as shown in Figure 2F), and this could well prove advantageous from an endosomal escape perspective (discussed later). As an approximation, there was about 50% of the sLDH in the liposome core and 50% on the liposome surface as well as in aqueous solution, determined by elemental analysis. In comparison, most L-LDH particles (>90%) were found in aqueous solution using the same procedure to make L-LDH–liposome.

### 3.2. Composite System Stability

**Figure 3:**
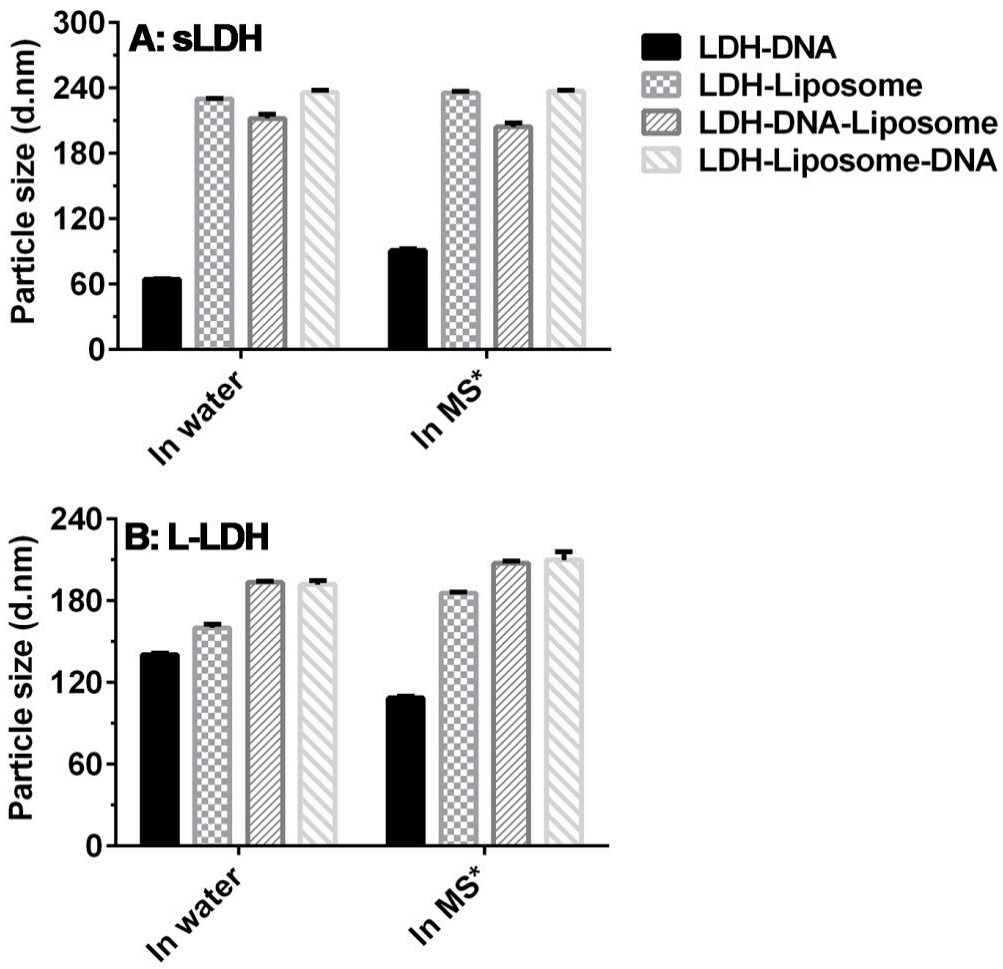
The *Z*-average particle sizes of LDH NPs and LDH–liposome formulations loaded with dsDNA (dsDNA = 10 µg/mL; the mass ratio LDH:dsDNA = 20:1; EPC:dsDNA = 20:1; * MS = medium + serum, *i.e.*, complete cell culture medium).

Figure 3A shows the particle sizes of sLDH and sLDH–liposome formulations loaded with dsDNA in water and in cell culture medium at a dsDNA concentration of 10 µg/mL. sLDH–dsDNA has a larger particle size in cell culture media (~90 nm) than in water (~64 nm), which can be attributed to the adsorption of serum proteins onto the surface of sLDH NPs. In contrast, the sLDH–liposome suspension and sLDH–liposome loaded with dsDNA possessed an average particle size between 200 and 240 nm, which is consistent with the size of cationic liposomes loaded with siRNA reported by Wu *et al.* [40], and this particle size range is also appropriate for subsequent cellular uptake. Moreover, this observation illustrates that loading dsDNA into the sLDH–liposome composite system does not significantly affect composite size.

### 3.3. Cytotoxicity of LDH and LDH–Liposome NPs

**Figure 4:**
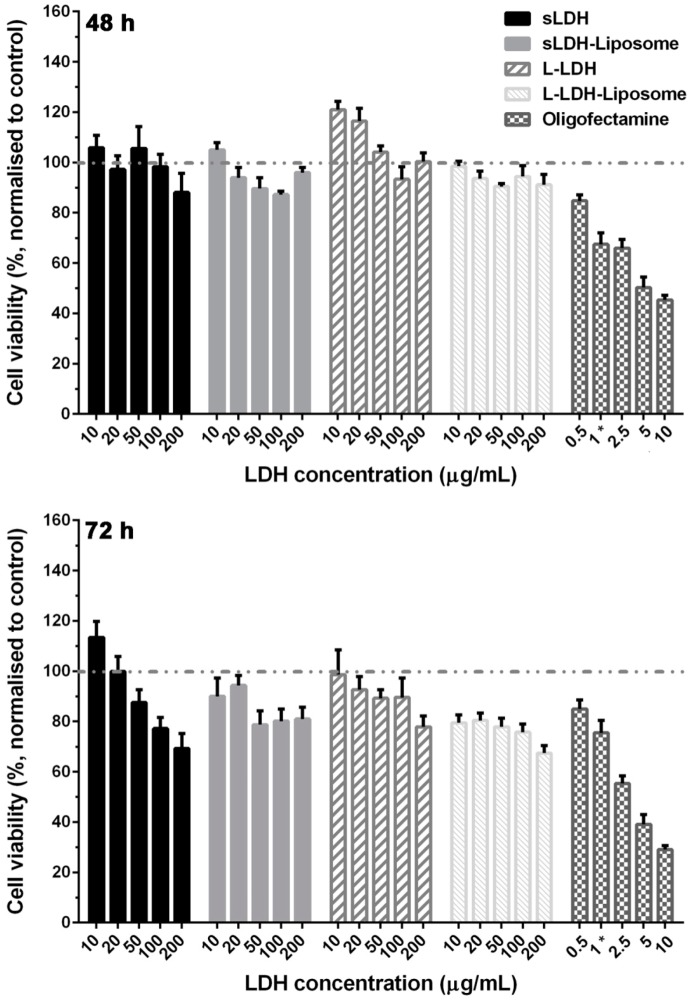
Cell viability of LDH and LDH–liposome NPs (MTT assay, in LDH–liposome formulations; LDH:EPC mass ratio = 1:1; transfection reagent Oligofectamine™ was used as the positive control; 1 * = minimum recommended dose of Oligofectamine™).

LDH and LDH-liposome NPs show negligible cytotoxicity at 72 h, even when employing artificially larger concentrations (*i.e.*, 100–200 µg/mL, >10-times the practical concentration), as shown in Figure 4, with >90% cell viability at 48 h and >70% cell viability at 72 h at 200 µg/mL of LDH in the systems. In contrast, transfection reagent Oligofectamine™ (Life Technologies, Carlsbad, CA, USA) exhibits significantly higher cytotoxicity at a comparable dose (~45% cell viability at 48 h and ~30% cell viability at 72 h, *p* < 0.0001). Consistent with previous reports, the cytotoxicity of LDH [17,38] and liposomes composed of neutral lipids [46,47] show moderate levels of cytotoxicity.

### 3.4. DNA Loading

**Figure 5:**
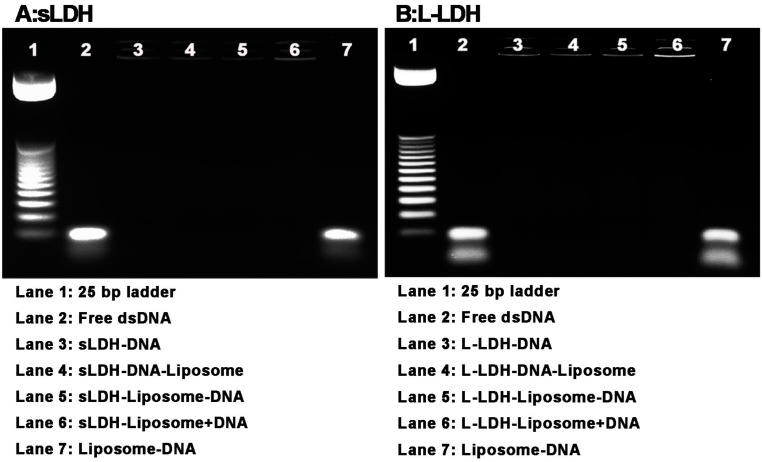
Electrophoresis mobility of the HFDM LDH–liposome composite for dsDNA loading (LDH:DNA mass ratio = 20:1; EPC:DNA mass ratio = 20:1).

Figure 5A shows the electrophoretic mobility of sLDH–liposome composites in the presence of dsDNA. It is evident that sLDH and sLDH–liposome composites completely bind dsDNA, irrespective of the dsDNA loading method (sLDH:dsDNA mass ratio = 20:1, Lane 3 to Lane 6), driven by the positively-charged property of sLDH NPs, which fully immobilises the negatively-charged DNA in the wells at this mass ratio. However, neutral liposomes (EPC:dsDNA = 20:1, Lane 7) cannot fully retard dsDNA migration in the well. This difference reveals, as alluded to earlier, that there are likely to be sLDH NPs residing outside of the sLDH–liposome composite, which help to immobilise dsDNA in the well (sLDH NPs fully associate dsDNA at a mass ratio of 5:1, as reported earlier [39]), as proposed and shown in Figure 2F.

L-LDH and L-LDH–liposome yielded similar profiles for dsDNA immobilisation, as shown in Figure 5B. L-LDH and all L-LDH–liposome formulations can fully complex with dsDNA in the wells regardless of the dsDNA loading method (Lane 3 to 6; positively-charged L-LDH NPs can also fully combine with DNA at this mass ratio). This also indicates that L-LDH NPs are almost entirely residing outside the L-LDH–liposome composites, thus allowing direct interaction with dsDNA. In addition, the two faintest bands in Lanes 2 and 7 (Figure 5B) arise from strand dissociation (to ssDNA), where there was not any LDH involved, which can occur upon extended storage of dsDNA.

### 3.5. Cellular Delivery

In consideration of the high cellular delivery of DNA using LDH NPs only (~100% of positive cells were observed when 40 nM of Cy3–DNA was used with LDH NPs), 20 nM of Cy3–DNA was used here to evaluate the cellular uptake differences between LDH and LDH–liposome composite.

**Figure 6:**
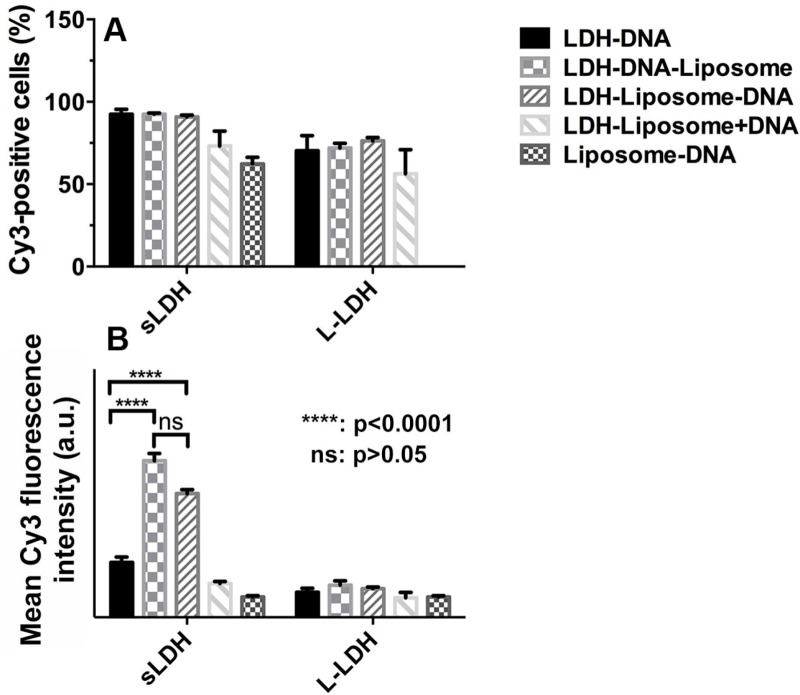
Cellular uptake of DNA–Cy3 by HCT-116 cells in terms of Cy3-positive cell percent (**A**) and mean Cy3-fluorescence intensity (**B**) (20 nM DNA, LDH:DNA mass ratio = 20:1, EPC:DNA = 20:1 and 4 h incubation.)

Cellular uptake of Cy3–DNA (20 nM) delivered using either LDH or LDH–liposome composite after 4 h of incubation are shown in Figure 6A,B. The LDH–liposome composite led to a similar number of Cy3-positive cell populations when compared to (s/L-)LDH alone. Unsurprisingly, the average amount of DNA delivered into each cell by sLDH–DNA–liposome and sLDH–liposome–DNA was 2.9- and 2.2-fold higher, respectively, when compared to that achieved with sLDH alone. In contrast, sLDH–liposome + DNA and liposome–DNA gave a lower relative average Cy3 intensity/cell (= Cy3 intensity of Cy3 positive cells × Cy3 positive cell percentage; the fluorescence of non-Cy3 positive cells was disregarded) than sLDH alone. The reason for the latter observation is most likely due to the lower loading efficiency of dsDNA into liposomes (Figure 5A), while for the former, this could be due to the competition for internalisation existing between empty sLDH–liposomes, blank liposomes and sLDH–DNA NPs; more likely, the encapsulated sLDH NPs are redundant in this case. The results indicate that the sLDH–DNA–liposome delivered the most DNA into HCT-116 cells, followed by sLDH–liposome–DNA. Clearly, sLDH–liposome + DNA delivered the least amount of DNA, for which one of the main reasons could be that most DNA is associated with the free sLDH in suspensions, and thus, DNA–sLDH is not hybridized with the liposome. Thus, sLDH–DNA–liposome and sLDH–liposome–DNA composites would be the better choice for future investigation.

The average Cy3 intensity delivered by L-LDH–liposome composites was much lower than that achieved by sLDH–liposome composites (Figure 6B). Moreover, their average Cy3 intensity is very similar in these cases, consistent with the idea that L-LDH largely resides outside the L-LDH–liposomes, which leads to a similar internalisation profile of DNA–Cy3 via L-LDH NPs.

### 3.6. Why does the sLDH–Liposome Composite Enhance Cellular Delivery?

sLDH–liposome composites possess improved cellular delivery properties, and this could be attributed in part to the encapsulation of sLDH NPs inside liposomal vesicles. Firstly, association of sLDH with liposomal vesicles reduces the risk of aggregation between sLDH NPs and serum proteins, thus improving the colloidal stability of sLDH(–DNA) NPs, so that the bioavailability and retention of sLDH–DNA NPs in the cell culture media is improved. The reduced protein binding was also reported by Yan *et al.* [37] for a PEGylated lipid-coated LDH delivery system. Other researchers in the field have also related the increased uptake/delivery of cell penetrating peptides, micelles-iron oxide NPs and LDH NPs to their greater stability under physiological conditions [37,48,49,50]. Moreover, the smaller dimensions of the resulting complexes normally lead to rapid, receptor-mediated internalisation, while larger, aggregated complexes may show less efficient unspecific uptake through adsorptive endocytosis, which is a relatively slower process [51]. Furthermore, with improved composite suspension stability, the system would be expected to perform well in both suspended and adherent cell lines (previously, we found that transfection of the suspended cell line CHO-S using LDH NPs was not very successful, unlike adherent cell lines, such as HEK293T, NIH 3T3, COS-7 and CHO-K1 [16]).

Aside from the composite size and colloidal stability, another key driver to internalisation, is the affinity of phospholipids (from liposomes) for lipids of the cell membrane [52,53]. This is also confirmed by the >50% of Cy3-positive cell population achieved with the liposome only, although gene loading efficiency by the liposome is probably only 50% (Figure 5, Lane 7).

**Figure 7:**
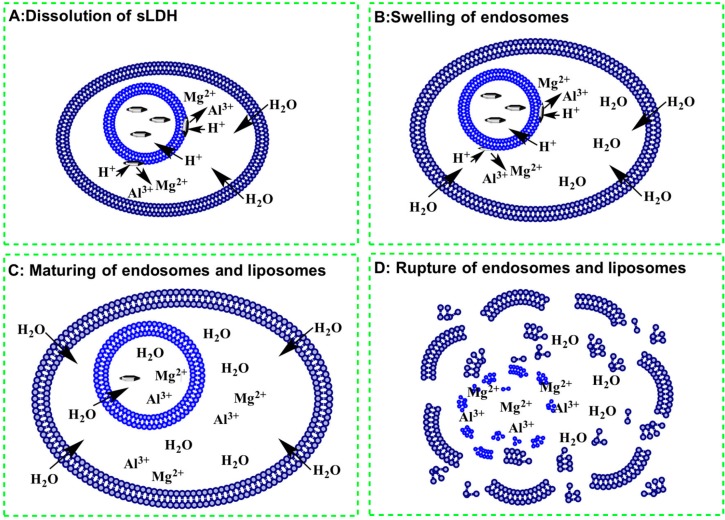
Possible endosomal escape pathway for sLDH–liposome composite.

Followed by enhanced cellular uptake, efficient cargo release is also crucial for any competent gene delivery vector. The proposed endosomal escape pathway for this sLDH–liposome composite is outlined in Figure 7 [21,22]. Following internalisation of the sLDH–liposome composite, sLDH NPs on the liposomal external surface dissolve gradually under the acidic conditions inside the endosomes. This results in increased osmotic pressure inside, causes an influx of water and then swells the endosome (Figure 7A,B). Further dissolution of sLDH NPs leads to further swelling of liposomal and endosomal vesicles (Figure 7C), finally rupturing the endosomes and liposomes to release their cargos into the cytoplasm (Figure 7D). Note that in the case of liposomes (neutral liposomes here), the internalised liposome–DNA complexes cannot escape from the endosome efficiently, like cationic liposomes or liposomes with disrupting agents [54,55,56,57], which might lead to the bulk of DNA being degraded in the lysosome.

## 4. Conclusions

LDH–liposome composites prepared using the HFDM method possess a good particle size distribution with a *Z*-average size of ~200 nm immediately after hydration, and they remain suitable for cellular uptake studies. The composite systems are stable in culture medium, with limited cytotoxicity observed. More interestingly, the sLDH–liposome system showed a higher cellular delivery efficiency (2–3-times higher) than sLDH alone, while the L-LDH–liposome composite did not exhibit significant differences from L-LDH. The reasons we put forward for these observation are that: (1) sLDH can be easily encapsulated in the aqueous core of the liposome because of its relatively smaller size; (2) some sLDH NPs could attach to the surface of the liposome bilayer, which helps bind to and protect DNA and, more importantly, helps in its escape from the endosome; (3) the hydrophobicity of liposomes may also facilitate their cellular uptake. Thus, this research has demonstrated that a carefully engineered combination of sLDH and liposome synergises cellular delivery efficiency, by taking advantage of both systems’ salient features.
